# A Case of Non-Arteritic Anterior Ischemic Optic Neuropathy with COVID-19

**DOI:** 10.7759/cureus.11950

**Published:** 2020-12-07

**Authors:** Jonathan Rho, Stephen C Dryden, Charles D McGuffey, Brian T Fowler, James Fleming

**Affiliations:** 1 Ophthalmology, Hamilton Eye Institute, Memphis, USA

**Keywords:** neuroophthalmology, naion, ophthalmology, neurology, ischemic optic neuropathy, vision loss, covid-19, optic neuropathy

## Abstract

Non-arteritic ischemic optic neuropathy (NAION) is thought to be caused by loss of blood flow to the optic nerve which in turn causes an acute, unilateral and painless vision loss that affects older vasculopathic patients. We report a case of a 43-year-old Hispanic male with the classic presentation of NAION in the setting of a coronavirus disease 2019 (COVID-19) infection. It is well documented that severe acute respiratory syndrome coronavirus 2 (SARS-CoV-2) can cause hypoxemia and thrombophilia in patients, both of which may contribute to the development of NAION. It is uncertain whether the association of NAION and COVID-19 was causal or coincidental but the purpose of this case report is to argue that there is biological plausibility and to help shed light on potential ophthalmologic complications of COVID-19.

## Introduction

Non-arteritic ischemic optic neuropathy (NAION) is presumed to be caused by lack of perfusion to the optic nerve and subsequently causes an acute, unilateral vision loss that is painless. This disease classically affects older patients with vasculopathic risk factors and sleep apnea. Patients may complain of altitudinal visual field defects but any type of visual field defect may be seen. Patients present with optic disc edema in the acute setting but subsequently develop optic atrophy that may be seen as a pale optic disc on examination. We present a case of a 43-year-old Hispanic male with NAION in the setting of coronavirus disease 2019 (COVID-19).

## Case presentation

A 43-year-old Hispanic male with a past medical history of diabetes (DM) and borderline hyperlipidemia (HLD) and no prior ophthalmic examination was referred to our institute due to sudden, painless inferior vision loss that he experienced upon awakening in the right eye two weeks prior to presentation. Four weeks prior to presentation, the patient tested positive for severe acute respiratory syndrome coronavirus 2 (SARS-CoV-2) by nasal swabbing with constitutional symptoms of fever, cough, and right-sided headache. At presentation, his viral symptoms had resolved except the inferior vision loss. He denied a history of snoring or apneic episodes while sleeping, jaw claudication, headache, scalp tenderness, fever, weight loss, muscle weakness, or eye pain. His social and ophthalmic history was unremarkable.

**Figure 1:**
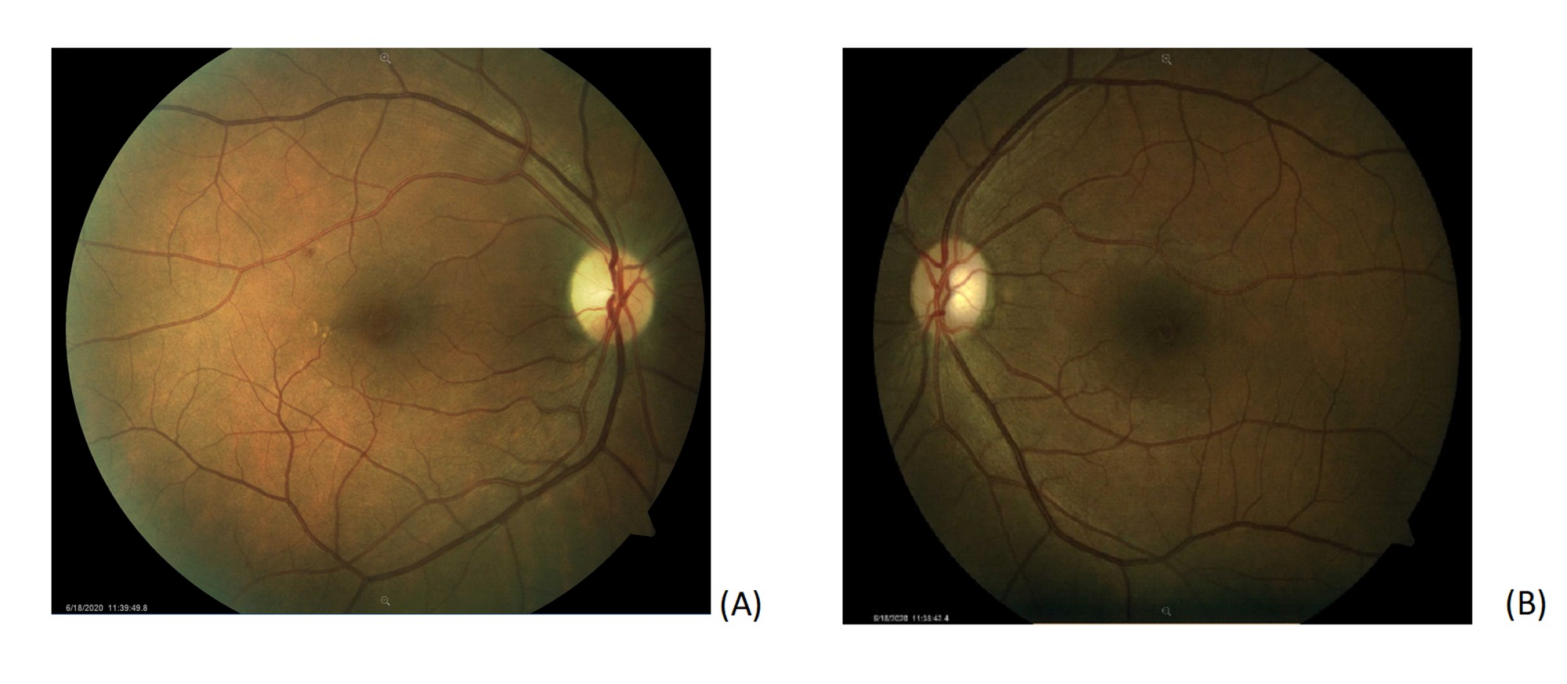
Color fundus photography of both eyes. (A) Fundus photo of the right eye showing a small microaneurysm with exudates along the superotemporal arcade without subretinal fluid and temporal pallor of the optic nerve. (B) Fundus photo of the left eye is normal with a cup-to-disk ratio of 0.3.

On examination, his best-corrected visual acuity (BCVA) was 20/30 OD and 20/20 OS by Snellen chart. Examination of the pupils revealed a relative afferent pupillary defect (RAPD) OD. Confrontational visual field demonstrated an inferior hemifield defect OD. Intraocular pressures, extraocular movements, and slit-lamp examination were normal OU. Dilated funduscopic examination (DFE) revealed a small microaneurysm with exudates along the superotemporal arcade without subretinal fluid and temporal pallor of the right optic nerve (Figure 1).

**Figure 2:**
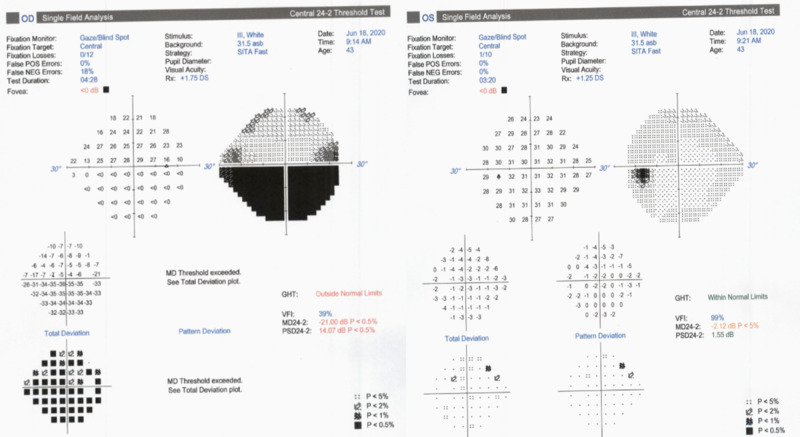
Humphrey Visual Field (HVF) 24-2 of both eyes. HVF OD is moderately reliable with 0/12 fixation losses, 0% false positive errors, and 18% false negative errors showing a dense inferior altitudinal defect that respects the horizontal meridian. HVF OS is reliable with 1/10 fixation losses, 0% false positive errors, and 0% false negative errors that does not show a specific visual loss pattern.

Humphrey visual field (HVF) 24-2 of the right eye revealed a dense inferior altitudinal defect that respects the horizontal meridian (Figure 2).

DFE and HVF 24-2 OS were normal. A retinal nerve fiber layer analysis (RNFL) of the right eye was thicker than left in all quadrants particularly superotemporally. Our patient had a body mass index (BMI) of 28.47 kg/m2, was normotensive and tested negative for hepatitis C and human immunodeficiency virus. A complete blood count, erythrocyte sedimentation rate, hemoglobin A1c (HbA1c), complete metabolic panel, prothrombin, partial thromboplastin time, antithrombin, protein C, protein S, lupus anticoagulant, and lipid profile all resulted within normal range except for HbA1c of 9.2% and borderline hyperlipidemia. Neuroimaging was not performed as the patient had no orbital signs on physical examination. We advised the patient to establish care with a primary care physician to optimize vasculopathic risk factors including hyperlipidemia and blood glucose and return to the clinic in six weeks for repeat DFE, RNFL, and HVF.

## Discussion

The combination of a patient with a history of DM presenting with a painless monocular inferior altitudinal defect upon wakening with optic nerve pallor is suspicious for NAION. Interestingly in our patient is the timing of NAION following COVID-19 infection. To our knowledge, this is the first case report of a patient who tested positive with SARS-CoV-2 with sequential COVID-19 symptoms and NAION.

NAION is thought to develop from circulatory insufficiency of the posterior ciliary arteries supplying the optic nerve, though the exact pathogenesis is uncertain. Some well-documented risk factors are having a small, crowded optic disc (disc at risk) or other vasculopathic risk factors. However, the association between NAION and hypercoagulability remains controversial [1]. A prospective cohort study by Kuhli-Hattenbach et al. found a strong association between early-onset NAION (age ≤55) and thrombophilic disorder (P=0.0002). The authors also report a link between the absence of cardiovascular risk factors in patients with NAION and thrombophilia (P=0.0059) [2].

It is well documented that SARS-CoV-2 can cause significant inflammation resulting in hypercoagulability - often manifesting as pulmonary embolism, deep-vein thrombosis, ischemic strokes, or myocardial infarcts [3]. There are some patients with COVID-19 that present with hypoxemia out of proportion to symptoms such as dyspnea, a phenomenon known as “silent hypoxemia”. Although respiratory pathophysiology can explain most of the findings of silent hypoxemia, it is still to be determined whether COVID-19 affects the carotid chemoreceptors resulting in a blunted dyspnea response [4]. In short, COVID-19 can cause clinically significant hypercoagulability and hypoxemia.

Since our patient had minimal cardiovascular risk factors and early onset NAION at the age of 43, he meets the characteristics described by Kuhli-Hattenbach et al. of patients suffering from NAION and thrombophilia [2]. Also, because the pathophysiology of NAION is circulatory insufficiency to the optic nerve, hypoxemia can exacerbate conditions for NAION. As mentioned, patients with COVID-19 infection can manifest with hypercoagulability and hypoxemia, both of which may contribute to the development of NAION. It is possible that our patient with DM was on the border of developing NAION and the complications of COVID-19 overwhelmed the auto-regulatory mechanisms of optic nerve perfusion, resulting in NAION. Currently, it is not certain whether the development of NAION and COVID-19 was causal or coincidental, but the goal of this case report is to argue that there is biological plausibility and to help shed light on potential ophthalmologic complications of COVID-19.

## Conclusions

NAION is presumed to be caused by lack of perfusion to the optic nerve. Our relatively younger patient with some vasculopathic risk factors suffered from NAION in the setting of a COVID-19 infection. COVID-19 may have caused a hypercoagulable and low oxygen state which may have contributed to the development of NAION. To our knowledge, this is the first reported case report of such relationship; however, more studies are needed to determine if this relationship is causal or coincidental.

## References

[1] Miller NR, Arnold AC (2015). Current concepts in the diagnosis, pathogenesis and management of nonarteritic anterior ischaemic optic neuropathy. Eye (Lond).

[2] Kuhli-Hattenbach C, Scharrer I, Lüchtenberg M, Hattenbach LO (2009). Selective thrombophilia screening of patients with nonarteritic anterior ischemic optic neuropathy. Graefes Arch Clin Exp Ophthalmol.

[3] Klok FA, Kruip MJHA, van der Meer NJM (2020). Incidence of thrombotic complications in critically ill ICU patients with COVID-19. Thromb Res.

[4] Tobin MJ, Laghi F, Jubran A (2020). Why COVID-19 silent hypoxemia is baffling to physicians. Am J Respir Crit Care Med.

